# Combination of Diabetes Risk Factors and Hepatic Steatosis in Chinese: The Cardiometabolic Risk in Chinese (CRC) Study

**DOI:** 10.1371/journal.pone.0090101

**Published:** 2014-03-03

**Authors:** Jun Liang, Yu Wang, Hongyan Li, Xuekui Liu, Qinqin Qiu, Lu Qi

**Affiliations:** 1 Department of Endocrinology, Xuzhou Central Hospital, Xuzhou Clinical School of Xuzhou Medical College, Xuzhou Institute of Medical Sciences, Xuzhou Institute of Diabetes, Affiliated Hospital of Southeast University, Xuzhou, Jiangsu, China; 2 Xuzhou Medical College, Xuzhou, Jiangsu, China; 3 Department of Nutrition, Harvard School of Public Health, Boston, Massachusetts, United States of America; 4 Channing Laboratory, Department of Medicine, Brigham and Women's Hospital and Harvard Medical School, Boston, Massachusetts, United States of America; University of Birmingham, United Kingdom

## Abstract

**Aims:**

Hepatic steatosis has been related to insulin resistance and increased diabetes risk. We assessed whether combination of diabetes risk factors, evaluated by the Finnish Diabetes Risk Score, was associated with risk of hepatic steatosis in an apparently healthy Chinese population.

**Research Design and Methods:**

The study samples were from a community-based health examination survey in central China. In total 1,780 men and women (18–64 y) were included in the final analyses. Hepatic steatosis was diagnosed by ultrasonography. We created combination of diabetes risk factors score on basis of age, Body Mass Index, waist circumference, physical activity at least 4 h a week, daily consumption of fruits, berries or vegetables, history of antihypertensive drug treatment, history of high blood glucose. The total risk score is a simple sum of the individual weights, and values range from 0 to 20.

**Results:**

Hepatic steatosis was present 18% in the total population. In multivariate models, the odds ratios of hepatic steatosis were 1.20 (95%CI 1.15–1.25) in men and 1.25 (95%CI 1.14–1.37) in women by each unit increase in the combination of diabetes risk factors score, after adjustment for blood pressure, liver enzymes, plasma lipids, and fasting glucose. The area under the receiver operating characteristic curve for hepatic steatosis was 0.78 (95%CI 0.76–0.80), 0.76 in men (95%CI 0.74–0.78) and 0.83 (95%CI 0.79–0.87) in women.

**Conclusions:**

Our data suggest that combination of major diabetes risk factors was significantly related to risk of hepatic steatosis in Chinese adults.

## Introduction

Non-alcoholic fatty liver disease (NAFLD) is the most common chronic liver disease in the world [1]–[3], which comprises a wide group of progressive alterations in liver structure and function, ranging from hepatic steatosis and non-alcoholic steatohepatitis (NASH) to fibrosis and cirrhosis [4].

In previous studies, it has been found that combination of diabetes risk factors, such as the Finnish Diabetes Risk Score (FINDRISC), was related to increased risk of hepatic steatosis [5]. However, these studies are mostly in the White population, and data in Asians are lacking. The present study was to evaluate whether combination of diabetes risk factors, evaluated as FINDRISC, was associated with hepatic steatosis in a large sample of Chinese adults.

## Methods

### 1 Study Population

In the Cardiometabolic Risk in Chinese (CRC) Study, we performed a community-based health examination survey for 6,431 individuals (18–93 y) who were randomly selected from residents living in the urban area of Xuzhou, China, in 2009. All subjects underwent a complete medical examination, a clinical consultation, blood laboratory tests, and an ultrasonographic abdominal scan. All individuals provided details of their demographic, medical history, and use of medication at the time of their clinical consultation. Individuals with missing data and those with a previous history of liver disease, defined as a positive test for hepatitis, history of cirrhosis, whose daily alcohol intake was> = 20 g, biliary disease, or diabetes mellitus were excluded from the present analysis. In total, 1,780 men and women (18–64 y) were included in the final analyses. There was not significant difference in age and anthropometrics between individuals who were included and those who were not included in the analyses. The protocol and informed consent document were approved by the ethics committee of the Central Hospital of Xuzhou, China. All patients gave written informed consent. Hepatic steatosis was diagnosed by ultrasonography using an abdominal probe at 2–5 MHz. Longitudinal, subcostal, ascending, and oblique scans were performed.

### 2 Anthropometric measures

Body weight was measured in light clothing to the nearest 0.1 kg and height to the nearest 0.5 cm. Height and body weight were measured with participants standing without shoes and heavy outer garments. Waist circumference was measured at the minimum abdominal girth to the nearest 0.1 cm. Body Mass Index(BMI) was calculated as weight (in kilograms) divided by height (in meters) squared. Blood pressure (BP) was measured after the subject had rested for at least 5 minutes with a mercury manometer by doctors. Three measurements, 60 seconds apart, were taken. The mean of the three measurements was used for analysis.

### 3 Assessment of biomarkers

Venous blood sample was drawn from all subjects after an overnight fast (10 h). The blood was transferred into glass tubes and allowed to clot at room temperature. Immediately following clotting serum was separated by centrifugation for 15 min at 3,000 rpm. Participants also underwent a 75-g oral glucose tolerance test (OGTT). The OGTT was carried out according to World Health Organization (WHO) recommendations. Blood samples were drawn at 120 minutes after the glucose or carbohydrate load. Plasma glucose was measured using the hexokinase glucose-6-phosphate dehydrogenase method (Type 7600; Hitachi Ltd, Tokyo, Japan). The levels of total cholesterol, triglyceride, high-density lipoprotein cholesterol (HDL-C), low-density lipoprotein cholesterol (LDL-C), aspartate aminotransferase(AST), Alanine aminotransferase(ALT), alkaline phosphatase, and gamma glutamyl transpeptidase (γGT) were determined enzymatically using an autoanalyzer (Type 7600; Hitachi Ltd., Tokyo, Japan). Fasting insulin was measured by a radioimmunoassay ethod (Pharmacia, Uppsala, Sweden). HbA1c was measured using high performance liquid chromatography (HPLC; HLC-723G7 hemoglobin HPLC ana-lyzer, Tosoh Corp.) according to the standardized method.

### 4 Combination of diabetes risk factors

As previously described [6], The FINDRISC contains seven questions, with categorized answers, about age, BMI, waist circumference, physical activity at least 4 h a week, daily consumption of fruits, berries or vegetables, history of antihypertensive drug treatment, history of high blood glucose. The total risk score is a simple sum of the individual weights, and values range from 0 to 20. The questionnaire was applied by registered nutritionists at the time of the routine diet interview of the health evaluation protocol.

### 5 Statistical analyses

Statistical analyses were performed by Statistical Package for Social Science (SPSS) version 13.0. Continuous variables were expressed in mean ± standard deviation. Two-sided t-tests and chi-square tests were used to analyze the differences between the groups at baseline. Student's t-test and one-way analysis of variance (ANOVA) were used to compare continuous variables. The relations between FINDRISC levels and hepatic steatosis were examined using logistic regression models, adjusting for covariates including BP, AST, ALT, gamma GT, total cholesterol, HDL cholesterol, fasting glucose. Odds ratios with 95% confidence intervals for the FINDRISC per unit increase for prediction of steatosis were performed by logistic regression. All reported P values are two tailed. The level of statistical significance was set to 0.05.

## Results

Among the 1780 participants, there were 932 (52.4%) men and 848 (47.6%) women. The baseline characteristics of the study population are presented in Table 1. Compared to women, men had a higher BMI, waist circumference, blood pressure, fasting plasma glucose, 2 h Plasma glucose, total serum cholesterol, LDL-C, and triglyceride (*P* values<0.001), whereas women had a higher mean HDL-C level compared with men. The mean FINDRISC was 8 in men and 6 in women. The prevalence of hepatic steatosis in this study population was 18.6%. There was no statistically significant difference between men and women, 19.5% in men, 17.4% in women.

**Table 1:** Clinical characteristics of the participants by gender.

	Female	Male	Total
N	848	1630	2478
Age (years)	44.4±7.8	46.4±9.3	45.7±8.8
BMI (kg/m2)	23.0±3.0	25.3±2.9	24.5±3.2
Waist circumference (cm)	78.3±8.1	89.8±8.2	85.9±9.8
Systolic blood pressure (mmHg)	116.1±14.9	126.4±15.4	122.9±16.0
Diastolic blood pressure (mmHg)	74.8±10.1	81.8±11.2	79.4±11.3
Fasting plasma glucose (mmol/L)	4.9±0.6	5.4±1.3	5.2±1.1
2 h Plasma glucose (mmol/L)	6.7±1.8	7.5±3.1	7.2±2.8
Fasting serum insulin (mmol/L)	8.3±4.6	9.8±7.0	9.3±6.3
Total cholesterol (mmol/L)	5.0±0.8	5.2±0.9	5.1±0.9
Serum Triglycerides (mmol/L)	1.1±0.8	2.0±1.8	1.7±1.6
Serum HDL cholesterol (mmol/L)	1.4±0.3	1.2±0.3	1.3±0.3
Serum LDL cholesterol (mmol/L)	2.9±0.7	3.0±0.8	3.0±0.8
Aspartate aminotransferase (mmol/L)	16.0±12.1	26.5±19.0	22.9±17.7
Alanine aminotransferase (mmol/L)	15.8±5.9	19.3±8.9	18.1±8.2
Alkaline phosphatase (mmol/L)	51.3±16.0	59.5±15.8	56.7±16.3
Gamma glutamyl transpeptidase (mmol/L)	17.2±18.3	39.0±40.1	31.6±36.3
FINDRISC	6.1±3.3	7.5±3.6	7.0±3.5
The prevalence of steatosis(n/N)	149/848	182/932	331/1780

Data are means ± standard deviations for the continuous variables and percentage for the categorical variables.

**Figure 1:**
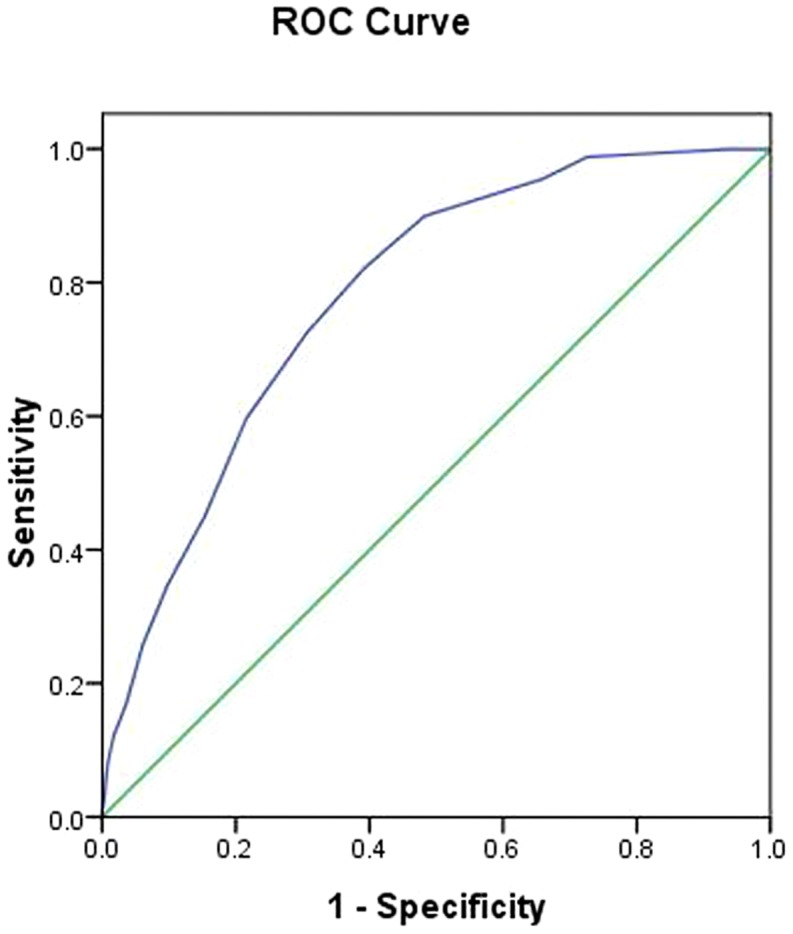
Receiver operating characteristic curve for the performance of steatosis in the study population. The analyses were adjusted for blood pressure, liver enzymes, plasma lipids, and fasting glucose.

The performance of combination of diabetes risk factors in predicting hepatic steatosis using receiver operating characteristic curve(ROC) analysis is presented in Figure 1. The area under the ROC curve for steatosis was 0.78 (95% CI 0.76–0.80), 0.76 in men (95% CI 0.74–0.78) and 0.83(95% CI 0.79–0.87) in women after adjustment for blood pressure, liver enzymes, plasma lipids, and fasting glucose. For cut point of FINDRISC > = 8, sensitivity was 0.70 and specificity was 0.72.

All the risk factors for cardiovascular disease had a strong direct association with the FINDRISC values(**Table 2**). Liver enzymes, such as aspartate aminotransferase, alanine aminotransferase, alkaline phosphatase and gamma glutamyl transpeptidase escalated with the increasing value of the risk score. In both men and women, there was a marked increase in the prevalence of steatosis with increasing value of the risk score. In men, the proportion of subjects who were classified as having the steatosis from 2% in the lowest risk score category to 62.1% in the highest. In women, the corresponding numbers were 1% and 58%.

**Table 2:** Cardiovascular risk factor profile by gender and FINDRISC values.

FINDRISC value						
Men						
	0–3	4–6	7–10	11–14	15–20	*P* value
Age (years)	39.4	44.9	47.6	51.2	55.3	<0.001
BMI (kg/m^2^)	22.6	24.4	25.7	27.6	29.2	<0.001
Waist circumference (cm)	82.5	86.5	91.5	96.7	100.0	<0.001
Systolic blood pressure (mmHg)	114.8	120.3	129.0	138.4	143.4	<0.001
Diastolic blood pressure (mmHg)	73.3	78.0	83.7	90.3	91.3	<0.001
Fasting plasma glucose (mmol/L)	4.9	5.1	5.3	5.9	7.5	<0.001
2 h Plasma glucose (mmol/L)	6.1	6.7	7.7	8.8	12.6	<0.001
Fasting serum insulin (mmol/L)	6.9	8.4	10.2	12.2	18.2	<0.001
Total cholesterol (mmol/L)	4.8	5.0	5.3	5.4	5.5	0.020
Serum Triglycerides (mmol/L)	1.2	1.7	2.1	2.5	3.1	<0.001
Serum HDL cholesterol (mmol/L)	1.2	1.2	1.2	1.1	1.1	<0.001
Serum LDL cholesterol (mmol/L)	2.9	3.0	3.1	3.1	3.0	<0.001
Aspartate aminotransferase (mmol/L)	19.9	25.0	28.2	30.3	31.3	<0.001
Alanine aminotransferase (mmol/L)	16.7	18.7	20.3	20.4	21.7	<0.001
Alkaline phosphatase (mmol/L)	57.5	59.6	59.6	59.4	66.3	<0.001
Gamma glutamyl transpeptidase (mmol/L)	25.0	35.7	42.6	47.6	53.1	<0.001
The prevalence of steatosis (n/N)	2/100	24/260	66/320	72/223	18/29	<0.001
women						
Age (years)	38.8	44.7	46.2	53.8	59.7	<0.001
BMI (kg/m^2^)	20.9	22.0	24.4	26.9	29.9	<0.001
Waist circumference (cm)	72.4	75.5	82.7	89.2	95.0	<0.001
Systolic blood pressure (mmHg)	108.7	111.9	120.4	134.1	149.7	<0.001
Diastolic blood pressure (mmHg)	70.1	72.3	77.9	85.5	90.0	<0.001
Fasting plasma glucose (mmol/L)	4.7	4.9	5.1	5.3	6.2	<0.001
2 h Plasma glucose (mmol/L)	6.1	6.5	7.0	7.7	9.7	<0.001
Fasting serum insulin (mmol/L)	7.3	7.1	9.2	12.0	13.6	<0.001
Total cholesterol (mmol/L)	4.7	5.0	5.1	5.3	5.6	<0.001
Serum Triglycerides (mmol/L)	0.8	1.0	1.3	1.6	1.7	<0.001
Serum HDL cholesterol (mmol/L)	1.5	1.4	1.3	1.3	1.2	<0.001
Serum LDL cholesterol (mmol/L)	2.6	2.8	3.1	3.2	3.5	0.023
Aspartate aminotransferase (mmol/L)	14.3	14.9	17.5	19.6	20.5	0.001
Alanine aminotransferase (mmol/L)	15.2	15.1	16.3	17.6	17.8	0.003
Gamma glutamyl transpeptidase (mmol/L)	14.9	15.1	19.9	21.2	25.5	0.001
The prevalence of steatosis (n/N)	3/245	24/249	46/270	24/72	7/12	<0.001

Data are means except where noted otherwise.

**Table 3** shows the association of the FINDRISC as a continuous variable with the presence of steatosis. In the unadjusted model each unit increase in the FINDRISC questionnaire increased the odds ratios of steatosis by 1.33 in men and 1.45 in women. This association remained robust even after adjustment for blood pressure, liver enzymes, plasma lipids, and fasting glucose, and the odds ratios were 1.20 in men and 1.25 in women. Model 1: FINDRISC only; Model 2: model 1 + SBP + DBP; Model 3: model 1 + AST, ALT, γGT; Model 4: model 1 + TC + HDL-C; Model 5: model 1 + FBG; Model 6: model 1 + SBP + DBP+ AST+ALT+γGT+ TC+ HDL-C + FBG.

**Table 3:** Odds ratios with 95% confidence intervals for the FINDRISC per unit increase for prediction of steatosis.

	Men	Women
	OR 95%CI	OR 95%CI
Model 1: FINDRISC only	1.33 1.28–1.38	1.45 1.34–1.57
Model 2: model 1 + SBP + DBP;	1.37 1.31–1.43	1.40 1.28–1.54
Model 3: model 1 + AST, ALT, γGT	1.32 1.27–1.37	1.45 1.34–1.57
Model 4: model 1 + TC+ HDL-C	1.28 1.24–1.33	1.35 1.24–1.47
Model 5: model 1 + FBG	1.34 1.29–1.39	1.39 1.28–1.51
Model 6: model 1 + SBP + DBP+ AST+ALT+γGT+ TC+ HDL-C + FBG	1.20 1.15–1.25	1.25 1.14–1.37

SBP: systolic blood pressure, DBP: diastolic blood pressure;

AST: aspartate aminotransferase, ALT: Alanine aminotransferase, γGT: gamma glutamyl transpeptidase;

TC: total cholesterol, HDL-C: high-density lipoprotein cholesterol;

FBG: fasting blood glucose;

CI: confidence intervals.

## Discussion

In the present study of a large sample of Chinese adults, we found that combination of diabetes risk factors, evaluated as the FINDRISC, was associated with increased risk of hepatic steatosis. The associations were consistent in men and women even after adjustment for blood pressure, liver enzymes, plasma lipids, and fasting glucose.

The prevalence of hepatic steatosis in this study population was 18.6%, 19.5% in men, 17.6% in women, which was lower than previous reports in other parts of the world (20–30% in Europe and the Middle East [7]–[9], 20–30% in North America and similar countries [10], [11]). Differences may relate to ethnicity and lifestyle, with the majority of our study subjects having lower proportion of men in our study (52.4%) than in Carvalho's study (80.8%). NAFLD was more frequent in men [12].

Finding effective approaches to prevent NAFLD is a critical public health priority. Given the recent clinical trials showing that prevention of NAFLD with lifestyle intervention is possible, there is also increasing interest in the development of tools to identify high-risk individuals who might benefit from interventions, or persons worth further testing for such as ultrasonography or liver biopsy. Given the expense, inconvenience, limitations, and risks associated with a liver biopsy, it is unsuitable for the screening of the general population for such a prevalent condition.

Several modalities have been used to diagnose NAFLD non-invasively with variable sensitivity and specificity, including ultrasonography, computed tomography (CT), magnetic resonance imaging (MRI), and MRS. Ultrasound is the most readily available imaging modality for the diagnosis of fatty liver. Ultrasound findings of NAFLD include hepatomegaly, diffuse increased echogenicity of the liver parenchyma, blurring of intrahepatic vessels, and loss of echoes of the posterior hepatic segments. A review of studies comparing the role of ultrasound in diagnosing histologically proven NAFLD reported an overall sensitivity and specificity for the detection of moderate to severe steatosis (> = 20–30% steatosis) of 84% and 93%, respectively, with an accuracy of 0.93 [13]. Ultrasound sensitivity varies considerably with liver fat content and to technical limitations in morbidly obese patients. In a recent meta-analysis of the performance of different imaging modalities in diagnosing biopsy-proven NAFLD, CT was equivalent if not inferior to ultrasound [14]. In addition, CT carries the disadvantage of radiation exposure. Magnetic resonance imaging and MRS perform better than ultrasound or CT, particularly in detecting lower degrees of steatosis. However, they are costly and not widely available.

The presence of NAFLD is correlated with the degree of peripheral and hepatic IR [15], [16]. Ultrasound-diagnosed NAFLD increased the risk of diabetes 2.5-fold [17]. The reverse is also true, with increases in fasting blood glucose and insulin levels leading to a higher overall prevalence of NAFLD, which is estimated to be 63–69% in patients with diabetes [18]–[20].

Since there is a close association of obesity, insulin resistance, and type 2 diabetes with NAFLD, we assessed whether combination of diabetes risk factors, evaluated as FINDRISC, was associated with hepatic steatosis in a large sample of Chinese adults. Risk factors for type 2 diabetes seem to differ between ethnic groups. Consistent in all of them, a family history of diabetes confers an increased risk of developing type 2 diabetes, but its relative effect decreases with increasing prevalence of type 2 diabetes in the population. Low level of physical activity has been associated with risk of diabetes. The incidence of type 2 diabetes in individuals with impaired glucose tolerance can be reduced through diet and exercise [21]. Other risk factors, like abdominal obesity, hypertension confers an increased risk of type 2 diabetes [22], [23].

Several previous studies have shown that FINDRISC was related to type 2 diabetes, the metabolic syndrome, insulin resistance [6], [24], [25]. In a recent cross-sectional study including 821 Brazil non-diabetic subjects without previous hepatic disease, it was found that the FINDRISC could be a useful primary screening tool for the presence of steatosis [5]. In that study the ROC curve for the undiagnosed prevalent diabetes was 0.80, 0.80 in men and 0.83 in women, which was consistent with the present findings.

In this study, we found that combination of diabetes risk factors, evaluated by the FINDRISC, was associated with risk of hepatic steatosis in an apparently healthy Chinese population. The area under the ROC curve was 0.78. For cut point of the sum of combination of diabetes risk factors score≥8, sensitivity is 0.70 and specificity is 0.72. In addition, the score was shown to be closely associated with various CVD risk factors. BMI and waist circumference performed worse than the FINDRISC in predicting steatosis in our study. FINDRISC can be applied by primary health care physicians and other health care professionals without the need of laboratory and/or imaging tests. Identified high-risk individuals should be evaluated by special tests. Also FINDRISC predicts not only steatosis but also type 2 diabetes, abnormal glucose tolerance, the metabolic syndrome [6], [12], [24], [25].

However, several limitations of this study warrant consideration. Firstly, in our study, we did not collection information of Quantitative alcoholic consumption and smoking. For example, failure to adjust for smoking may lead to over-estimation of relationship between FINDRISC and liver steatosis. Cigarette smoking has been consistently related to T2DM risk [26]. If smoking are adjusted, the resulting OR and ROC values tend to be lower. Secondly, ultrasound could have under-estimated the already high prevalence of steatosis in our population. Several studies have shown that ultrasound for detecting hepatic steatosis has a sensitivity of 60% to 94%, and a specificity of 84% to 95%. Its sensitivity is reduced in the morbidly obese, and its performance is highly operator-dependent. The prevalence of liver steatosis among Chinese population is 20.09% [27], while the prevalence of hepatic steatosis in this study population was 18.6%. Lastly, the study was performed in a Chinese population. Further studies in other populations of different ethnicities are warranted to verify our findings.

In summary, in the present study of Chinese adults, combination of diabetes risk factors was associated with hepatic steatosis, and it also could be a useful primary screening tool for the presence of steatosis.
